# Explaining the Association Between Urbanicity and Psychotic-Like Experiences in Pre-Adolescence: The Indirect Effect of Urban Exposures

**DOI:** 10.3389/fpsyt.2022.831089

**Published:** 2022-03-11

**Authors:** Abhishek Saxena, David Dodell-Feder

**Affiliations:** ^1^Department of Psychology, University of Rochester, Rochester, NY, United States; ^2^Department of Neuroscience, University of Rochester Medical Center, Rochester, NY, United States

**Keywords:** psychotic-like experiences, psychosis, urbanicity, poverty, deprivation, pollution, pre-adolescence

## Abstract

Urban living is a growing worldwide phenomenon with more than two-thirds of people expected to live in cities by 2050. Although there are many benefits to living in an urban environment, urbanicity has also been associated with deleterious health outcomes, including increased risk for psychotic outcomes particularly when the urban exposure occurs in pre-adolescence. However, the mechanisms underlying this association is unclear. Here, we utilize one-year follow-up data from a large (*N*=7,979), nationwide study of pre-adolescence in the United States to clarify why urbanicity (i.e., census-tract population density) might impact psychotic-like experiences (PLE) by looking at the indirect effect of eight candidate urbanicity-related physical (e.g., pollution) and social (e.g., poverty) exposures. Consistent with other work, we found that of the evaluated exposures related to urbanicity, several were also related to increased number of PLE: PM_2.5_, proximity to roads, census-level homes at-risk for exposure to lead paint, census-level poverty, and census-level income-disparity. These same urban-related exposures were also related to the persistence of PLE after 1 year, but not new onset of PLE. Mediation analysis revealed that a substantial proportion the urbanicity-PLE association (number and persistence) could be explained by PM_2.5_ (23–44%), families in poverty (68–93%), and income disparity (67–80%). Together, these findings suggest that specific urban-related exposures contribute to the existence and maintenance, but not onset of PLE, which might help to explain why those in urban environments are disproportionately at-risk for psychosis and point toward areas for public health intervention.

## Introduction

City living is an increasing societal phenomenon. The World Health Organization estimates that 68% of the world's population will reside in urban areas by the year 2050, compared to 55% of the population in 2018 (1). Those migrating to urban environments often do so to for better access to employment opportunities, education, health care, public utilities, among other benefits (2–4). While city living offers many social, economic, and health benefits, it also has significant drawbacks in these areas, making it critical to understand the potential net consequences of increasing global urbanization (3, 4). Of particular interest has been the impact of urbanicity on mental health. An already robust and growing body of retrospective and prospective research has shown that urban (as opposed to rural) birth, upbringing, or living increases risk for psychosis (5–12), and does so in a dose-response manner (13, 14). Meta-analytic estimates have shown that the risk for psychosis approximately doubles in urban environments (15–18), and population-based cohort studies have shown that urbanicity explains 30% or more of incident cases (8, 9), making the impact of urbanicity on psychosis risk as large as or larger than other known non-genetic environmental risk factors (19). Importantly, the association between urbanicity and psychosis remains after controlling for many potential confounding factors, including drug use and genetic risk, (16, 17, 20, 21), and also cannot be explained by selective migration (22). Other studies have demonstrated that changes in urbanicity prospectively predict changes in psychosis risk later in life (7, 14). Thus, together, the available data suggest that the association between urbanicity and psychosis could be causal in nature (17, 23), perhaps by way of an interaction between urbanicity and constitutional vulnerability factors (e.g., genetic vulnerability, birth complications) (17, 24).

Despite the clear etiological importance of the urban environment for psychosis, one of the most critical issues related to this association remains poorly understood: what is the mechanism through which urbanicity impacts risk? Urban environments involve a wide variety of physical (e.g., pollution) and social (e.g., poverty) exposures. It may be these exposures that explain, in part, why urban environments impact psychosis risk (20, 25, 26). To this point, a variety of urban-related physical exposures have been shown to increase risk for and exacerbate psychotic symptoms including air pollution (27–31), and xenobiotic heavy metal (i.e., lead) exposure (32–34). Similarly, many urban-related social exposures have been associated with psychosis risk and exacerbation, including socioeconomic disparities (35–37), exposure to violence and trauma (38, 39), and exposure to social exclusion, discrimination, and racism due to having an ethnic, migrant, sexual, or gender minority group membership (40–42).

When taken together, the possibility that the urban environment may impact psychosis risk indirectly through a variety of pathways seems strong. In line with this idea, some of the urban-related exposures have been shown to mediate the relation between urbanicity and psychosis risk in both child and adult samples. For example, on physical exposures, various air pollutants and level of traffic have been shown to mediate or attenuate the association between urbanicity and psychosis (43, 44). Similarly, for social exposures, such as low social cohesion and crime victimization, have been shown to moderate the relationship between childhood and adolescent psychotic symptoms and urbanicity (10, 45). In similar studies, the impact of racial/ethnic minority status (the hypothesized exposure being racism and discrimination) on increased psychosis risk remains even when controlling for the effect of urbanicity (13). Further, some of these findings have been shown to be specific to psychosis over other mental health outcomes (10, 45). Despite these findings, the literature on how urban exposures may mediate the urbanicity-psychosis risk association is limited. On this point, to our knowledge, there are no studies evaluating the indirect effect of both physical and social exposures in the same study.

Thus, the current study aims to further our understanding of the urbanicity-PLE association in pre-adolescence by evaluating whether urbanicity, defined by census-tract population density (people per km^2^), impacts PLE through a suite of candidate physical and social urban-related exposures in a large, nationally representative pre-adolescent sample. In doing so, we aimed to better understand the mechanisms through which urbanicity relates to PLE. Evaluating these issues in late childhood and early adolescence is critical given that this represents the developmental period where urbanicity seems to impact psychosis risk (7, 14), as well as when many of the urban-related exposures (e.g., lead) may be most potent in their impact [e.g., (27, 46)]. Further, urban living is associated with increased PLE in childhood and early adolescence (47) as well as the persistence of PLE in adulthood (48) making early life urbanicity an important marker of concurrent and prospective psychosis risk.

To address these aims, we utilized baseline and one-year follow-up data from the Adolescent Brain Cognitive Development (ABCD) study (49); abcdstudy.org; a longitudinal, nation-wide study on the development of nearly 12,000 children across the United States. This study follows children starting at ages nine and ten, and includes information on child psychotic-like experiences (50, 51) (PLE), as well as demographic, social, and geolocation data (52, 53). By using the ABCD dataset, we are able to explore the associations between urbanicity, candidate urban-related exposures, and later PLE. Further, in line with other work (10, 43), we are able to test the hypothesis that urban-related exposures mediate the urbanicity-PLE association. A recent study examined similar associations in the ABCD dataset, as well as the mediating role of brain volume using all baseline (i.e., cross-sectional) data (54). Importantly, here, our main goal was to investigate the mediating role of urban-related exposures (instead of brain volume), and to do so using one-year follow-up PLE data, which allowed us to look at longitudinal changes in PLE as a function of urbanicity and urban-related exposures. First, we evaluated whether urbanicity was associated with later PLE in pre-adolescence, as well as the specificity of this association by evaluating urbanicity's association with later externalizing and internalizing symptoms. Second, we evaluated whether urbanicity was associated with a broad range of candidate physical (i.e., air pollution, lead exposure), and social exposures (i.e., socioeconomic disadvantage) available in the ABCD dataset. Third, we evaluated whether those exposures related to urbanicity were also related to later PLE. Finally, we evaluated whether urbanicity was related to PLE by way of urban-exposures; that is, whether the physical and social urban exposures that were related to urbanicity and PLE, mediated the urbanicity-PLE association.

## Methods

### Participants

Data were obtained from participants in the Adolescent Brain Cognitive Development (ABCD) study; a longitudinal study following children beginning at ages nine- and ten-years from 21 research sites across the United States. The sociodemographic makeup of the sample closely matches that of the United States as a whole, with a slight oversampling of Black/African American and other race children that corresponds to a slight under-sampling of White and Hispanic children (49). We used baseline and one-year follow-up data from the ABCD Data Release 3.0, which was accessed through the National Institute of Health Data Archive (https://nda.nih.gov/abcd). Of the 11,878 participants who contributed baseline data, 11,235 participants (95%) contributed one-year follow-up data. We then removed 3,256 participants who were missing data on one or more of the baseline or one-year follow-up variables described below, leaving a final sample of 7,979 participants with complete datasets (Table 1). The final sample matched the excluded sample on all demographics except for race, where a greater proportion of data was missing for Black respondents and a lesser proportion of data was missing for White respondents (Table S1).

**Table 1:** Participant characteristics and variable descriptives.

**Variable**	***M* (*SD*) or *N* (%)**
**Demographics**	
Age (years)	9.91 (0.63)
Sex-at-birth	
Female	3,786 (47)
Male	4,193 (53)
Race/ethnicity	
Asian	171 (2)
Black	1,001 (13)
Hispanic	1,606 (20)
Other	827 (10)
White	4,374 (55)
Familial risk for psychosis^a^	
Yes	161 (2)
No	7,818 (98)
**Psychopathology**	
Prodromal questionnaire-brief child, one-year follow-up	
Total score	1.84 (3.14)
Total persistence	2,951 (37)
Total onset	873 (11)
Child behavior checklist, one-year follow-up	
Externalizing symptoms (T score)	45.29 (10.11)
Internalizing symptoms (T score)	48.17 (10.47)
**Urbanicity and candidate mediators**	
Urbanicity (population density; people per km^2^)	2,181.81 (2,610.97)
Average levels of PM_2.5_ in 2016 (μg/m^3^)	7.52 (2.55)
3-Year Average of ground-level NO_2_ (μg/m^3^)	2.45 (1.64)
Proximity to major roads (meters)	1,218.37 (1,323.29)
Homes at risk for exposure to lead-based paint (%)	20.50 (15.86)
Families below poverty line (%)	11.13 (11.99)
Income disparity^b^	2.09 (1.33)
Total adult violent offenses	3,619.72 (7,769.44)
Marijuana sales	471.49 (902.02)

a *Defined by caregiver report as to whether either biological parent “ever had a period lasting six months when they saw visions or heard voices or thought people were spying on them or plotting against them”*.

b *log of 100 x ratio of households with $50,000 in annual income (55)*.

### Measures

#### Urbanicity

Consistent with other work [e.g., (11)] urbanicity was defined as census-tract population density (persons per km^2^) at baseline. At baseline, respondents were able to identify up to six current residential addresses and the percentage of time they spent at each address. The ABCD dataset provides geolocation data for only current addresses and not previous addresses. For geolocation data to be appended to the ABCD dataset, respondents had to provide valid addresses. The ability to identify multiple residential addresses, allowed respondents to provide all the residences at which the pre-adolescent spent significant time. This takes into account pre-adolescents who, for example, have parents living separately, or who spend a significant time with a caregiver that is not their primary caregiver (e.g., grandparents, extended family, etc.). However, only one valid address was available for almost all (99%) included respondents, with the remaining offering two. Using the available valid addresses, a single score was created by weighting each residential address population density value by the percentage of time spent at that address. For respondents who accounted for more or less than 100% of their time across all usable addresses, a simple average was used.

#### Psychotic-Like Experiences and Psychopathology

PLE were assessed with the Prodromal Questionnaire-Brief Child Version [PQ-BC; (50)] completed at the 1-year follow-up. The PQ-BC is a 21-item self-report questionnaire modified and validated for children from the Prodromal Questionnaire-Brief (56) that assess a range of PLE such as unusual thought content, suspiciousness/persecutory ideas, grandiosity, and perceptual aberrations (50). Participants first indicated whether they had experienced the PLE. Those who indicated that they had experienced the PLE then indicated whether the PLE was bothersome. For each PLE that was identified as bothersome, participants rated how bothersome the experience was using a 1 to 5 visual response scale with higher scores denoting higher distress. Following other studies using the PQ-BC (50, 51, 57), total score and distress scores were used. The total score was calculated by summing the number of items endorsed (possible score: 0–21). The distress score was calculated by weighting the total score by the level of distress. A score of 0 indicates no PLE endorsed, 1 indicates one PLE endorsed with no distress, and scores of 2–6 indicate that a PLE was endorsed with some distress (possible score: 0-126). Prior work has demonstrated the PQ-BC to exhibit construct validity and adequate psychometric properties (50, 51). Because findings with the total and distress score were similar, and the total score more closely resembles the PLE score derived in other similar studies evaluating the impact of urbanicity on PLE through urban-related exposures (10, 43), we focused on the total score in the main text. Analyses with the distress score are provided in the supplement.

Following the analytical approach of others examining changes in PLE in the ABCD dataset (58), we created dichotomous outcome variables representing (a) the persistence of PLE from baseline to one-year follow-up and (b) the onset of new PLE at one-year follow-up. These variables were created by first evaluating the presence of any PLE at baseline (0 = no, 1 = yes) and follow-up (0 = no, 1 = yes). PLE persistence and onset were calculated such that persistence indicates the presence of at least one PLE at both baseline and follow-up (baseline = 1, follow-up = 1), and onset indicates the lack of any PLE at baseline and presence of at least one PLE at follow-up (baseline=0, follow-up=1). Analyses utilizing persistence or onset of PLE-associated distress are reported in the supplement.

To assess the specificity of the association between urbanicity and later PLE, we evaluated externalizing and internalizing symptoms using the respective broad band scores from the Child Behavior Checklist [CBCL; (59)]. The externalizing broad-band score indexes rule-breaking and aggressive behaviors, while the internalizing broad-band score indexes anxiety, depressed symptoms, and somatic complaints.

#### Candidate Mediators

Candidate mediators were selected from the baseline ABCD dataset if the variable (1) in theory related to city living, (2) was associated with increased risk for PLE or SSDs, and (3) in theory could explain the urbanicity-PLE association. Our selection of variables was such that the candidate mediators were physical (e.g., pollution) or social (e.g., violence) in nature, allowing us to test the impact of two classes of urban-associated exposures with potentially distinct mechanisms. We note that there were several other variables in the ABCD dataset that met these criteria but were not included either because other (non-composite) variables more directly indexed the exposure of interest (e.g., assessing families in poverty vs. area deprivation) or due to extremely low base rates (e.g., using marijuana sales vs. drug use). We identified 8 such candidate mediators (described below), with scores calculated using the same weighting procedure as for calculating urbanicity.

We included three measures of census-area air pollution: (1) annual average particulate matter with a diameter of 2.5 micrometers or less (PM_2.5_) levels for 2016 at 10 x 10 km^2^, (2) three-year ground-level average of nitrogen dioxide (NO_2_) at 10 x 10 km^2^, and (3) proximity to major roads, in meters. Both PM_2.5_ and NO_2_, have shown a relationship with higher incidence of psychotic disorders (27) and PLE (43), and with worsening psychotic symptoms (29, 30, 60–62). Similarly, proximity to major roads was used as a measure of traffic-related pollution, which is also associated with schizophrenia risk (44). We included one measure of census-area lead exposure: the estimated percentage of houses at-risk for lead exposure due to lead paint, which also has been linked to psychosis risk (32–34). Finally, using data from the US Federal Bureau of Investigation's Uniform Crime Reports, we included the number of arrests for marijuana sales in a census area as a proxy for use and exposure to marijuana, which also has been implicated as a component cause of psychosis (63–65).

We included two measures of census-area economic adversity: (1) the percentage of families below the poverty line (“families in poverty”), and (2) income disparity as defined by Singh (55) as the log of 100 x ratio of households with $50,000 in annual income). Both factors have been linked to the incidence and symptoms of psychosis (66–69). On social adversity, we included the total number of adult violent offenses in a participant's census-area as crime victimization has been shown to prospectively increase risk for PLE (10).

### Data Analysis

#### Associations Between Urbanicity, Psychopathology, and Candidate Urban-Exposures

Planned analyses were preregistered on the Open Science Framework (https://osf.io/rdsuj). Data analysis was performed using R (70). Following the analysis strategy of Newbury et al. (10), we first evaluated the association between urbanicity and psychopathology 1 year after baseline. PLE outcomes included PLE total score, persistence of PLE (dichotomous), and onset of PLE. We also examined associations between urbanicity and externalizing and internalizing symptoms to test for specificity. Second, we examined the association between urbanicity and the 8 identified exposures that may account for the association between urbanicity and psychopathology. Third, we examined the association between psychopathology and those exposures that we found were related to urbanicity.

In order to account for the nested structure of the data whereby the participant was nested within family and site, we used linear mixed-effects models for continuous outcome variables and generalized mixed-effects models for dichotomous outcome variables using the *lme4* package (71). These models included random intercepts for family and site. Results are reported as standardized beta coefficients for continuous outcomes and odds ratios for dichotomous outcomes along with 95% CIs, *p* values, and false-discovery rate (FDR) adjusted *p* values corrected for each group of tests. Associations are considered unexpected under the null hypothesis when FDR-adjusted *p* < 0.05. Analyses included age, sex, and familial risk for the relevant form of psychopathology outcome (i.e., psychosis, externalizing disorders, or internalizing disorders) as covariates.

A goal of this study was to maximize comparability with prior studies [e.g., (10, 43, 54)] thus, similar to those studies, we did not include race and ethnicity as covariates. Due to historical and current systematic racism in the United States, there is a disproportionate representation of racial and ethnic minorities in urban areas and disproportionate experiences with adverse urban-related exposures. This confounding would have made it difficult to tease apart the impact of race/ethnicity vs. the putative PLE risk factors investigated here. However, for those interested, sensitivity analyses where race/ethnicity are included as covariates in statistically significant mediation models are available in the supplement (Tables S4, S5).

#### Mediation Analyses

**Figure 1:**
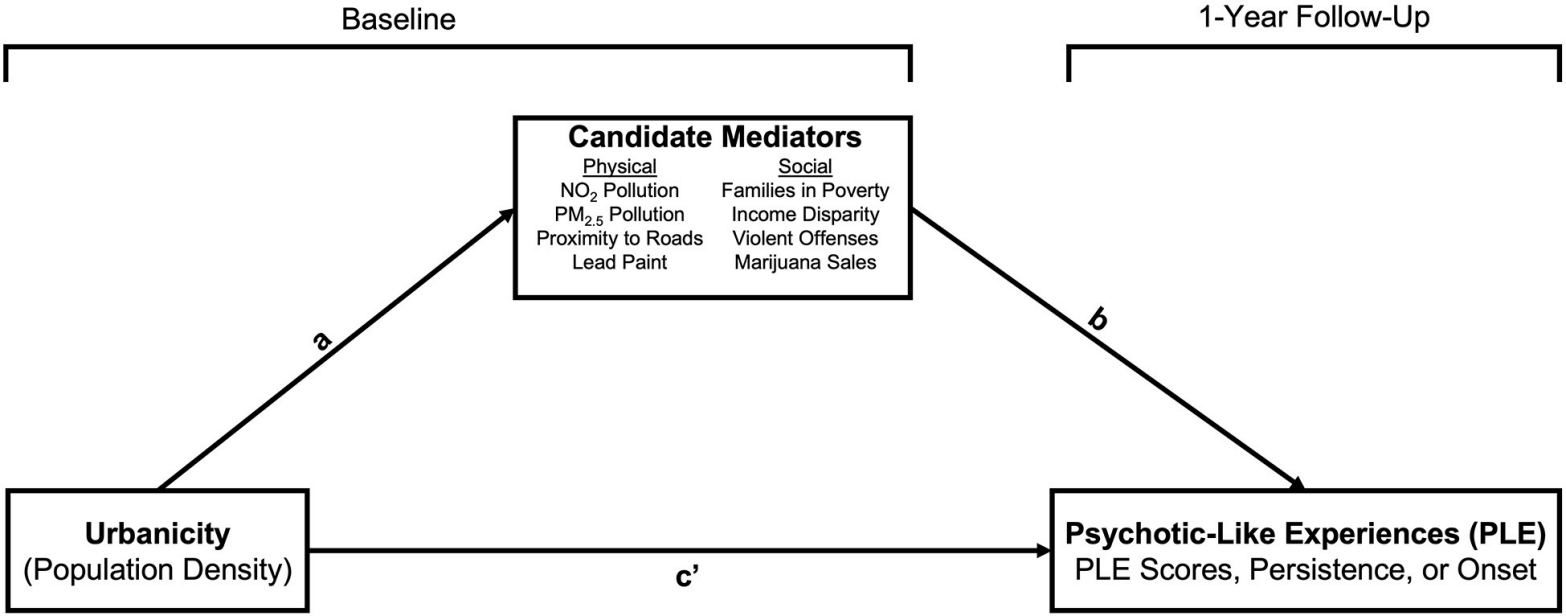
Mediation model. Age (in months) and sex-at-birth (M>F) were included as covariates on paths a and b.

Lastly, of the exposures that were found to be associated with urbanicity and PLE, we evaluated whether they accounted for the urbanicity-PLE association; that is, whether these exposures mediated the relation between urbanicity and PLE. In order to simplify models and avoid non-convergence, we split the sample by family, creating one dataset (*N* = 6,775) which contained only one child from families in which more than one child from the family was represented. Because our hypotheses concerned level 1 (i.e., within-person) effects, and all variables were measured at level 1, we conducted a single-level mediation model using the *lavaan* package (72), and accounted for the clustering of data due to site by generating cluster-robust parameter estimates for continuous PLE outcomes. *lavaan* does not support cluster-robust parameter estimate analyses for categorical outcomes so the mediation models using persistence and onset as the outcomes did not take into account the nesting of data within site. We used a Monte Carlo test of mediation (73, 74) using the *semTools* package (75) by evaluating the 95% CI generated from 50,000 simulated values of the indirect effect. We similarly included age, sex, and familial risk for psychosis as covariates in these models. As mentioned, sensitivity analyses with race/ethnicity as covariates are available in the supplement (Tables S4, S5). As a measure of effect size, we provide the absolute proportion mediated (76), which denotes the proportion of the total effect that can be attributed to the indirect effect [i.e., a^*^b/(a^*^b+c); Figure 1].

## Results

### Urbanicity and Psychopathology

First, we evaluated whether similar to other reports, greater urbanicity was associated with greater later-PLE in a pre-adolescent sample. In line with this other work, greater urbanicity was associated with more PLE measured one-year later (Table 2). On the longitudinal nature of the association, we found that greater urbanicity was associated with PLE persistence, but not PLE onset. PLE distress variables showed the same pattern. Since PLE total onset was not associated with urbanicity, this variable was dropped from further analysis.

**Table 2:** Associations between urbanicity (predictor), psychopathology, and candidate mediators.

**Outcome variable**	**β (*SE*) / Odds Ratio**	**95% CI**	***p***
**Psychopathology**			
Ple-total score	0.053 (0.013)	0.028, 0.078	<0.001^*^
Ple-total persistence	1.089	1.029, 1.151	0.003^*^
Ple-total onset	0.987	0.908, 1.074	0.765
Externalizing symptoms	0.011 (0.012)	−0.013, 0.036	0.364
Internalizing symptoms	0.018 (0.012)	−0.007, 0.042	0.160
**Candidate mediators**			
PM_2.5_	0.115 (0.006)	0.103, 0.127	<0.001^*^
NO_2_	0.219 (0.006)	0.207, 0.230	<0.001^*^
Proximity to roads	−0.117 (0.012)	−0.142, −0.093	<0.001^*^
Lead paint	0.316 (0.012)	0.293, 0.338	<0.001^*^
Families in poverty	0.324 (0.013)	0.299, 0.350	<0.001^*^
Income disparity	0.325 (0.012)	0.301, 0.350	<0.001^*^
Violent offenses	0.047 (0.003)	0.042, 0.053	<0.001^*^
Marijuana sales	0.038 (0.003)	0.032, 0.045	<0.001^*^

*Associations were corrected for effect of age, sex-at-birth, parental risk for psychosis and include the random intercepts for family nested in site*.

* *False Discovery Rate (FDR) corrected p < 0.05*.

To evaluate the specificity of the association between urbanicity and PLE in pre-adolescence, we also tested the association between urbanicity and other forms of psychopathology, namely, externalizing and internalizing symptoms. Neither externalizing nor internalizing symptoms were associated with urbanicity (Table 2). These variables too were dropped from further analyses.

### Urbanicity and Candidate Urban Exposures

Next, we evaluated which of the exposures selected were in fact concurrently related to urbanicity in our sample. All exposures were associated with urbanicity in the expected directions with the magnitude of association being largest with homes at risk for exposure to lead paint, families below the poverty line, and income disparity (Table 2).

### PLE and Urban Exposures

Next, we evaluated the association between later PLE and all of the exposures that were linked to urbanicity. PLE total was associated with, in order of increasing magnitude, proximity to roads, homes at risk for exposure to lead paint, PM_2.5_, families in poverty, and income disparity, all in the expected direction (Table 3). While PLE total was also associated with NO_2_, this association did not survive FDR-correction, and as a result was not included in further analyses. The same pattern of findings was found for PLE total persistence.

**Table 3:** Associations between candidate mediators and PLE-total outcomes.

	**Total score**			**Total persistence**		
**Variable**	**β (*SE*)**	**95% CI**	***p***	**Odds ratio**	**95% CI**	***p***
PM_2.5_	0.072 (0.021)	0.029, 0.113	0.001^*^	1.228	1.112, 1.356	<0.001^*^
NO_2_	0.043 (0.021)	0.001, 0.085	0.046	1.099	0.996, 1.213	0.061
Proximity to roads	−0.029 (0.012)	−0.052, −0.006	0.014^*^	0.942	0.893, 0.994	0.028^*^
Lead paint	0.045 (0.013)	0.020, 0.070	0.001^*^	1.069	1.009, 1.132	0.023^*^
Families in poverty	0.105 (0.012)	0.081, 0.128	<0.001^*^	1.246	1.180, 1.316	<0.001^*^
Income disparity	0.116 (0.012)	0.092, 0.140	<0.001^*^	1.251	1.183, 1.322	<0.001^*^
Violent offenses	0.031 (0.030)	−0.029, 0.092	0.301	1.092	0.938, 1.270	0.257
Marijuana sales	0.039 (0.028)	−0.017, 0.096	0.171	1.119	0.973, 1.287	0.116

*Associations were corrected for effect of age, sex-at-birth, parental risk for psychosis and include the random intercepts for family nested in site*.

* *False Discovery Rate (FDR) corrected p < 0.05*.

### The Impact of Urbanicity on PLE Through Urban-Related Exposures

Finally, we evaluated whether the association between urbanicity and later PLE could be explained by urban exposures; specifically, those exposures that were independently associated with both urbanicity and later PLE in our sample (specifically, PLE total score and PLE total persistence). The association between each PLE outcome and urbanicity were tested with the five previously identified urban-related exposures: PM_2.5_ pollution, proximity to roads, homes at-risk for lead paint, families in poverty, and income disparity. Monte Carlo mediation tests of the indirect effect demonstrated that the association between urbanicity and PLE total was mediated by PM_2.5_, accounting for 23.0% of the association, families in poverty, accounting for 67.7% of the association, and income disparity, accounting for 66.6% of the association (Table 4). Similarly, the association between urbanicity and PLE total persistence was mediated by PM_2.5_, accounting for 44.1% of the association, families in poverty, accounting for 93.0% of the association, and income disparity, accounting for 79.8% of the association. Neither proximity to roads nor homes at-risk for lead paint significantly mediated the association between PLE outcome and urbanicity. Sensitivity analyses in which race and ethnicity were included as covariates demonstrated that the magnitude of the mediations were generally reduced (Tables S4, S5). PM2.5 mediated both PLE total score and PLE total persistence. Income disparity only significantly mediated PLE total persistence. Families in poverty did not mediate either PLE total score or PLE total persistence. We note however that each racial and ethnic minority group was strongly associated with urbanicity and all three candidate mediators, suggesting that race and ethnicity variables acted as confounds.

**Table 4:** Mediation analysis testing indirect effects of urbanicity on psychotic-like experiences.

	**Total Score**, **β** **[95% CI]**				**Total persistence, odds ratio [95% CI]**			
**Mediator**	**Total**	**Direct**	**Indirect**	**Proportion mediated**	**Total**	**Direct**	**Indirect**	**Proportion mediated**
PM_2.5_	0.045 [−0.012, 0.103]	0.035 [−0.023, 0.092]	**0.010 [0.000, 0.028]**	0.230	1.040 [1.011, 1.069]	1.022 [0.993, 1.051]	**1.017 [1.012, 1.023]**	0.441
Proximity to roads	0.045 [−0.012, 0.102]	0.043 [−0.013, 0.100]	0.002 [−0.003, 0.007]	0.040	1.040 [1.011, 1.069]	1.038 [1.009, 1.067]	1.002 [0.997, 1.006]	0.041
Lead paint	0.045 [−0.012, 0.103]	0.040 [−0.018, 0.098]	0.005 [−0.009, 0.021]	0.112	1.040 [1.011, 1.069]	1.037 [1.008, 1.067]	1.002 [0.993, 1.012]	0.058
Families in poverty	0.045 [−0.015, 0.101]	0.015 [−0.033, 0.062]	**0.031 [0.008, 0.060]**	0.677	1.040 [1.011, 1.069]	1.003 [0.975, 1.031]	**1.037 [1.028, 1.045]**	0.930
Income disparity	0.057 [−0.009, 0.118]	0.027 [−0.027, 0.082]	**0.030 [0.008, 0.057]**	0.666	1.040 [1.011, 1.069]	1.007 [0.979, 1.035]	**1.037 [1.029, 1.045]**	0.798

*Bolded values indicate a significant indirect effect*.

## Discussion

In this study, we aimed to better understand the nature of the association between urbanicity and PLE in a large, nation-wide pre-adolescent sample. Consistent with other work (18), we found that greater urbanicity was associated with greater number of PLE and associated distress measured one-year later. In contrast, we found no association between urbanicity and externalizing or internalizing symptoms, suggesting some specificity of the impact of urbanicity on mental health outcomes in pre-adolescence. Of those physical and social exposures we found to be related to urbanicity, only certain exposures—PM_2.5_, proximity to roads, houses at risk for lead paint, families in poverty, and income disparity—were related to the number of PLE assessed one-year later and persistence of PLE one year later. These variables were not related to the onset of PLE. These findings converge with other work showing PLE and SSD risk in children and adults is related to pollution (27, 28, 43), and living in countries or neighborhoods characterized by economic inequality and disadvantage (66–68). These findings also build upon prior cross-sectional analyses of urbanicity, urban-related exposures, and PLE in the ABCD dataset. Specifically, a prior study of baseline data revealed that a variety of environmental risk factors, including correlates of urbanicity, were associated with concurrent PLE, and that cortical volume partially mediated some of these relationships such as poverty, lead risk, and perception of neighborhood safety; as found in (54). Our study expands on that analysis by demonstrating that urbanicity has a significant longitudinal relationship with PLE.

Given the inter-relation between these variables, a key aim of this study was to evaluate the hypothesis that urbanicity is associated with PLE because of the impact of urban-related exposures. Partially consistent with this idea, we found that PM_2.5_, families in poverty, and income disparity mediated the association between urbanicity and number of PLE. Importantly, these variables explained a substantial proportion of the urbanicity-PLE association, ranging from 23.0% for PM_2.5_ to over 66% for families in poverty and income disparity. Further, these same exposures mediated the association between urbanicity and the one-year persistence of PLE, with exposures explaining an even greater proportion of the association, ranging from 44.1% for PM2.5 to over 79% for families in poverty and income disparity. In line with other work (10, 43), these findings suggest that it may not be urbanicity *per se* that impacts psychosis risk, but certain environmental attributes that covary with and perhaps result from urban environments.

Future work should investigate the processes through which pollution and economic inequality produces PLE, associated distress, and if the associations are causal in nature at all. Prior work has demonstrated that air pollution is associated with neuroinflammation, microglial activation, white matter deficits, and cognitive disturbances (77–80). These neurobiological changes are also associated with psychosis-risk states (81–83). On socio-economic disadvantage, poverty and inequality may promote unhealthy social comparisons related to social power and rank, which may promote views of oneself as a social subordinate or outsider; which are experiences connected to paranoia (84, 85). Socio-economic disadvantage may also erode social cohesion (i.e., interpersonal connectedness, shared values, mutual trust) and increase social disorganization (i.e., mobility, isolation, lack of social control, high crime); phenomena that also have been linked to increased PLE in childhood and adolescence (10, 45), and psychotic disorders in adulthood (35, 36, 66, 86, 87). Many of these experiences would fit within a social defeat model (42). Specifically, socio-economic disadvantage, and the consequences we speculate about above, promote the marginalization and subordination of disadvantaged individuals, leaving them socially isolated and more vulnerable to stress. Whatever the mechanisms, taken with other work, our findings suggest that urbanicity may set the physical and social context for experiences that are linked with increased PLE risk (26). It is notable and disconcerting that the association between these exposures and PLE are already present in late childhood.

The fact that the impact (presence of PLE and distress) of these exposures may occur as early as pre-adolescence highlights the importance of early prevention. Most psychosis-risk research has focused on older adolescents and young adults, which provides a short period for prevention given the modal age range of onset for psychotic spectrum disorders (88). Given that the association between well-established psychosis risk factors and PLE can be detected well before, it is important to consider the potential impact of intervening even earlier, and doing so by taking a public health-oriented approach that focuses on preventing the adverse exposures—like poverty and pollution—as opposed to the disorders themselves (89). Such interventions could include universal access to primary care, debt relief, or relocation to sustainable and green housing (90, 91). These actions could, in theory, reduce risk for psychosis and other health conditions impacted by the social and physical environment, as well as uplift underserved populations.

Several limitations are notable. First, we measured the association between urbanicity, urban-related exposures, and later PLE on a short timescale. It may be that some exposures require more time to impact PLE and mediate the urbanicity-PLE. Second, the short timescale may also explain the null results for PLE onset findings since few participants experienced an onset of PLE. It may be the case that some of these urban-related exposures are in fact related to PLE onset, but additional longitudinal data at a longer timescale would be needed to address this possibility. Third, many cases of childhood PLE do not persist (92). This leaves open the possibility that the urban exposures tested here may not predict PLE later in development. Fourth, it remains unclear the role these mediators may play in explaining the transition of PLE to psychotic illness vs. simply the persistence of PLE. Finally, we selected only a subset of all possible urban-related exposures available in the ABCD dataset that may explain the urbanicity-PLE association. Those tested here by no means provide an exhaustive account nor do they necessarily represent the most accurate way of assessing a given exposure (e.g., marijuana sales as a proxy for exposure to and use of marijuana). Similarly, some of the urban-related exposures are closely associated with other exposures not tested here that may also have an impact on PLE. For example, in addition to being a proxy for pollution, proximity to major roads may also be associated with noise pollution, which has been connected to schizophrenia in a work context (93, 94), cognitive impairment (95), and behavioral problems in children (96).

In summary, we find that many physical and social urban-related exposures are related to PLE numbers and distress measured one-year later. Further, a small number of these exposures—i.e., pollution, living in a high-poverty area, income disparity—explain, in part, the association between urbanicity and PLE in pre-adolescence, accounting for a substantial proportion of the total effect of urbanicity on PLE outcomes. Taken with other studies (10, 43), these findings help to explain why urbanicity has an impact on PLE, and intimate certain mechanisms that may be at work, which can explicitly be tested for in the future. The implications of such may be increasingly important as more of the global population moves into cities.

## Data Availability Statement

Publicly available datasets were analyzed in this study. This data can be found here: https://dx.doi.org/10.15154/1519352; NIMH Data Archive (Adolescent Brain Cognitive Development Study).

## Ethics Statement

The studies involving human participants were reviewed and approved by University of Rochester Research Subjects Review Board. Written informed consent to participate in this study was provided by the participants' legal guardian/next of kin.

## Author Contributions

AS and DD-F contributed to all aspects of this work, with DD-F also providing supervision. All authors contributed to the article and approved the submitted version.

## Funding

This work was supported indirectly by a grant from the National Institute of Mental Health (1L30MH117569-01 to DD-F).

## Conflict of Interest

The authors declare that the research was conducted in the absence of any commercial or financial relationships that could be construed as a potential conflict of interest.

## Publisher's Note

All claims expressed in this article are solely those of the authors and do not necessarily represent those of their affiliated organizations, or those of the publisher, the editors and the reviewers. Any product that may be evaluated in this article, or claim that may be made by its manufacturer, is not guaranteed or endorsed by the publisher.
