# Supplementary material for: Explaining the Association Between Urbanicity and Psychotic-Like Experiences in Pre-Adolescence: The Indirect Effect of Urban Exposures
Source: Front Psychiatry. 2022 Mar 11;13:831089. doi: 10.3389/fpsyt.2022.831089 (PMC8962621; doi:10.3389/fpsyt.2022.831089)
Supplement: Supplementary file 1 [file Data_Sheet_1.docx]

**Supplementary Materials**

Explaining the association between urbanicity and psychotic-like experiences in adolescence: The indirect effect of urban exposures

Supplemental Table 1. Participant Characteristics for Included and Excluded Respondents (p. 2)

Supplemental Table 2. Associations between PLE-Distress Variables and Urbanicity or Candidate Mediators (p. 3)

Supplemental Table 3. PLE-Distress Mediation Analyses (p. 4)

Supplemental Table 4. Race/Ethnicity Sensitivity Analyses for PLE-Total Mediation Analyses (p. 5)

Supplemental Table 5. Race/Ethnicity Sensitivity Analyses for PLE-Distress Mediation Analyses (p. 6)

**Supplemental Table 1**

*Participant Characteristics for Included and Excluded Respondents*

| **Variable** | **Included (N = 7979)** | **Excluded (N = 3899)** | **Difference Test** |
| --- | --- | --- | --- |
|  | ***M* (*SD*) or *N* (%)** | ***M* (*SD*) or *N* (%)** |  |
| ***Demographics*** |  |  |  |
| Age (years) | 9.91 (.63) | 9.92 (.62) | *t*(7749.1) = -0.600, *p* = .946 |
| Sex-at-Birth |  |  |  |
| Female | 3,786 (47) | 1,896 (49) | χ^2^(1) = 1.410, *p* = .235 |
| Male | 4,193 (53) | 2,003 (51) |  |
| Race/Ethnicity |  |  |  |
| Asian | 171 (2) | 81 (2) | χ^2^(4) = 0.854, *p* < .001 |
| Black | 1,001 (13) | 783 (20) |  |
| Hispanic | 1,606 (20) | 805 (21) |  |
| Other | 827 (10) | 420 (11) |  |
| White | 4,374 (55) | 1,808 (46) |  |
| Familial Risk for Psychosis^a^ |  |  |  |
| Yes | 161 (2) | 80 (2) | χ^2^(1) = 1.018, *p* = .313 |
| No | 7818 (98) | 3,312 (98) |  |

^a^N = 3392; Defined by caregiver report as to whether either biological parent “ever had a period lasting six months when they saw visions or heard voices or thought people were spying on them or plotting against them.”

**Supplemental Table 2**

*Associations between PLE-Distress Variables and Urbanicity or Candidate Mediators*

|  | **Distress Score** | | | **Distress Persistence** | | | **Distress Persistence** | | |
| --- | --- | --- | --- | --- | --- | --- | --- | --- | --- |
| **Variable** | **β (*SE*)** | **95% CI** | ***p*** | **Odds Ratio** | **95% CI** | ***p*** | **Odds Ratio** | **95% CI** | ***p*** |
| Urbanicity | .052 (.013) | .027, .077 | <.001* | 1.089 | 1.029, 1.151 | .002* | 1.016 | 0.943, 1.093 | .682 |
| NO_2_ | .038 (.020) | -.002, .078 | .063 | 1.186 | 1.066, 1.319 | .002* | - | - | - |
| PM_2.5_ | .053 (.020) | .013, .092 | .010* | 1.134 | 1.02, 1.261 | .020* | - | - | - |
| Proximity to Roads | -.023 (.012) | -.046, .000 | .047 | 0.96 | 0.903, 1.02 | .187 | - | - | - |
| Lead Paint | .039 (.013) | .014, .065 | .002* | 1.072 | 1.004, 1.143 | .036* | - | - | - |
| Families in Poverty | .094 (.012) | .070, .117 | <.001* | 1.191 | 1.12, 1.267 | <.001* | - | - | - |
| Income Disparity | .102 (.012) | .078, .126 | <.001* | 1.195 | 1.127, 1.268 | <.001* | - | - | - |
| Violent Offenses | .034 (.026) | -.018, .087 | .201 | 1.12 | 0.967, 1.298 | .131 | - | - | - |
| Marijuana Sales | .038 (.025) | -.011, .089 | .133 | 1.137 | 0.99, 1.307 | .070 | - | - | - |

Note: Associations were corrected for effect of age, sex-at-birth, parental risk for psychosis and include the random intercepts for family nested in site.

*False Discovery Rate (FDR) corrected *p* < .05.

Except for proximity to roads, later PLE distress score was associated with the same candidate mediators as the PLE total variables (PM_2.5_, houses at risk of exposure to lead paint, families in poverty, and income disparity). PLE distress persistence showed a slightly different pattern and was associated with PM_2.5_, NO_2_, families in poverty, and income disparity.

**Supplemental Table 3**

PLE-Distress Mediation Analyses

|  | **Distress Score, β [95% CI]** | | | | **Distress Persistence, Odds Ratio [95% CI]** | | | |
| --- | --- | --- | --- | --- | --- | --- | --- | --- |
| **Mediator** | **Total** | **Direct** | **Indirect** | **Proportion Mediated** | **Total** | **Direct** | **Indirect** | **Proportion Mediated** |
| PM_2.5_ | .049  [-.003, .101] | .041  [-.012, .094] | **.008  [.000, .022]** | .156 | 1.046  [1.013, 1.08] | 1.033  [1.000, 1.066] | **1.013  [1.008, 1.019]** | .291 |
| NO_2_ | - | - | - | - | 1.046  [1.013, 1.08] | 1.045  [1.009, 1.082] | 1.001  [0.983, 1.019] | .021 |
| Lead Paint | .049  [-.004, .101] | .045  [-.009, .099] | .004  [-.008, .018] | .072 | - | - | - | - |
| Families in Poverty | .049  [-.006, .100] | .021  [-.022, .063] | **.028  [.007, .054]** | .575 | 1.046  [1.013, 1.08] | 1.020  [0.987, 1.053] | **1.026  [1.016, 1.036]** | .568 |
| Income Disparity | .059  [-.001, .114] | .033  [-.016, .080] | **.026  [.007, .050]** | .556 | 1.046  [1.013, 1.08] | 1.028  [0.996, 1.060] | **1.027  [1.017, 1.036]** | .527 |

*Note.* Bolded values indicate a significant indirect effect.

The association between urbanicity and later PLE distress was mediated by families in poverty, accounting for 57.5% of the association, and income disparity, accounting for 55.6% of the association. Relatedly, the association between urbanicity and PLE distress persistence was mediated by PM_2.5_, accounting for 29.1% of the association, families in poverty, accounting for 56.8% of the association, and income disparity, accounting for 52.7% of the association.

**Supplemental Table 4**

Race/Ethnicity Sensitivity Analyses for PLE-Total Mediation Analyses

|  | **Total Score, β [95% CI]** | | | | **Total Persistence, Odds Ratio [95% CI]** | | | |
| --- | --- | --- | --- | --- | --- | --- | --- | --- |
| **Mediator** | **Total** | **Direct** | **Indirect** | **Proportion Mediated** | **Total** | **Direct** | **Indirect** | **Proportion Mediated** |
| PM_2.5_ | .005  [-.032, 0.042] | -.004  [-.041, .032] | **.010**  **[.002, .021]** | .696 | 0.993  [0.959, 1.028] | 0.975  [0.941, 1.010] | **1.018**  **[1.013, 1.024]** | .415 |
| Families in Poverty | .005  [-.033, .041] | -.001  [-.040, .036] | .007  [-.004, 0.020] | .832 | 0.993  [0.958, 1.028] | 0.987  [0.953, 1.022] | 1.006  [0.999, 1.012] | .306 |
| Income Disparity | .018  [-.028, .063] | .012  [-.034, .058] | .006  [-.005, .019] | .688 | 1.000  [0.965, 1.035] | 0.993  [0.958, 1.028] | **1.007**  **[1.000, 1.014]** | .355 |

*Note.* Bolded values indicate a significant indirect effect. Additional race/ethnicity covariates include Black, Hispanic, Asian, and Other Race, such that White pre-adolescents are the reference group.

**Supplemental Table**

Race/Ethnicity Sensitivity Analyses for PLE-Distress Mediation Analyses

|  | **Distress Score, β [95% CI]** | | | | **Distress Persistence, Odds Ratio [95% CI]** | | | |
| --- | --- | --- | --- | --- | --- | --- | --- | --- |
| **Mediator** | **Total** | **Direct** | **Indirect** | **Proportion Mediated** | **Total** | **Direct** | **Indirect** | **Proportion Mediated** |
| PM_2.5_ | .012  [-.021, .044] | .005  [-.028, .038] | **.007**  **[.000, .015]** | .563 | 1.002,  [0.963, 1.042] | 0.988,  [0.950, 1.028] | **1.014**  **[1.009, 1.020]** | .548 |
| Families in Poverty | .012  [-.022, .044] | .005  [-.028, .038] | .007  [-.003, .019] | .555 | 1.002,  [0.963, 1.043] | 0.992,  [0.953, 1.032] | **1.011**  **[1.003, 1.018]** | .565 |
| Income Disparity | .025  [-.017, .066] | .019  [-.022, .060] | .006  [-.004, .018] | .759 | 1.016,  [0.979, 1.055] | 1.008,  [0.971, 1.047] | **1.008**  **[1.001, 1.015]** | .637 |

*Note.* Bolded values indicate a significant indirect effect. Additional race/ethnicity covariates include Black, Hispanic, Asian, and Other Race, such that White pre-adolescents are the reference group.
